# Thermogenic adipocytes promote HDL turnover and reverse cholesterol transport

**DOI:** 10.1038/ncomms15010

**Published:** 2017-04-19

**Authors:** Alexander Bartelt, Clara John, Nicola Schaltenberg, Jimmy F. P. Berbée, Anna Worthmann, M. Lisa Cherradi, Christian Schlein, Julia Piepenburg, Mariëtte R. Boon, Franz Rinninger, Markus Heine, Klaus Toedter, Andreas Niemeier, Stefan K. Nilsson, Markus Fischer, Sander L. Wijers, Wouter van Marken Lichtenbelt, Ludger Scheja, Patrick C. N. Rensen, Joerg Heeren

**Affiliations:** 1 Department of Biochemistry and Molecular Cell Biology, University Medical Center Hamburg-Eppendorf, Martinistr. 52, 20246 Hamburg, Germany; 2 Department of Orthopaedics, University Medical Center Hamburg-Eppendorf, Martinistr. 52, 20246 Hamburg, Germany; 3 Department of Genetics and Complex Diseases and Sabri Ülker Center, Harvard T.H. Chan School of Public Health, 665 Huntington Avenue, Boston, Massachusetts 02115, USA; 4 Division of Endocrinology and Einthoven Laboratory for Experimental Vascular Medicine, Department of Medicine, Leiden University Medical Center, P.O. Box 9600, 2300 RC Leiden, The Netherlands; 5 III. Department of Internal Medicine, University Medical Center Hamburg-Eppendorf, Martinistr. 52, 20246 Hamburg, Germany; 6 Department of Medical Biosciences and Physiological Chemistry, Umeå University, Umeå 90787, Sweden; 7 Hamburg School of Food Science, Institute of Food Chemistry, University of Hamburg, Grindelallee 117, 20146 Hamburg, Germany; 8 Department of Human Biology, NUTRIM - School for Nutrition, Toxicology and Metabolism, Maastricht University Medical Center, Maastricht 6200 MD, The Netherlands

## Abstract

Brown and beige adipocytes combust nutrients for thermogenesis and through their metabolic activity decrease pro-atherogenic remnant lipoproteins in hyperlipidemic mice. However, whether the activation of thermogenic adipocytes affects the metabolism and anti-atherogenic properties of high-density lipoproteins (HDL) is unknown. Here, we report a reduction in atherosclerosis in response to pharmacological stimulation of thermogenesis linked to increased HDL levels in APOE*3-Leiden.CETP mice. Both cold-induced and pharmacological thermogenic activation enhances HDL remodelling, which is associated with specific lipidomic changes in mouse and human HDL. Furthermore, thermogenic stimulation promotes HDL-cholesterol clearance and increases macrophage-to-faeces reverse cholesterol transport in mice. Mechanistically, we show that intravascular lipolysis by adipocyte lipoprotein lipase and hepatic uptake of HDL by scavenger receptor B-I are the driving forces of HDL-cholesterol disposal in liver. Our findings corroborate the notion that high metabolic activity of thermogenic adipocytes confers atheroprotective properties via increased systemic cholesterol flux through the HDL compartment.

## Abstract (web summary)

Activation of brown adipose tissue (BAT) reduces the development of atherosclerosis in animal models. Here the authors show that BAT activation also increases reverse cholesterol transport and turnover of high-density lipoprotein, which likely contributes to the anti-atherosclerotic effect of BAT activation.

Brown adipose tissue (BAT) is the primary organ for heat production in small mammals in response to cold; BAT is also present and active in humans^1^,^2^,^3^,^4^,^5^,^6^,^7^. While cold is the natural stimulus^8^, thermogenic adipocytes can also be activated by treatment with selective β3-adrenergic receptor agonists such as CL316,243 (CL) in both mice and humans^9^,^10^. It is well accepted that thermogenic adipocytes contribute to energy expenditure in rodents and humans^11^,^12^,^13^,^14^,^15^,^16^, although the relative contribution is larger in rodents. Nevertheless, the high metabolic activity of BAT^17^,^18^,^19^ and adipose tissue browning, referring to the formation of so-called beige adipocytes in white adipose tissue (WAT)^17^,^20^,^21^, suggest that the activation of brown and beige adipocytes may be successfully targeted to combat metabolic diseases in humans. Apart from glucose, fatty acids are the main fuel for the metabolic activity of BAT. We have shown that BAT activity controls plasma levels of triglyceride (TG)-rich lipoproteins (TRLs), both by selective uptake of TRL-derived fatty acids that are liberated from TG by lipoprotein lipase (LPL) as well as uptake of whole TRLs^22^,^23^.

Previously, we demonstrated that the activation of thermogenic adipocytes reduces cholesterol levels and protects from atherosclerosis in transgenic mice expressing both a loss-of-function variant of human apolipoprotein E (APOE*3-Leiden; E3L) and the human cholesteryl ester transfer protein (E3L.CETP mice)^9^. This mouse model, unlike apolipoprotein E-deficient mice or low-density lipoprotein receptor-deficient mice^24^, responds well to the lipid-lowering effects of thermogenic activation either with cold or CL^9^. However, while the role of pro-atherogenic cholesterol-rich TRL remnants in this context is well-understood^9^, whether thermogenic adipocytes modulate the metabolism of HDL is unclear. This is of importance, as HDL particles are key components in the process of removing excess cholesterol from cells in peripheral organs and possibly from macrophages in atherosclerotic lesions^25^,^26^. HDL represents a special class of lipoproteins with a variety of biological activities^27^,^28^. The most broadly studied function of HDL is facilitating cholesterol efflux from cells and driving macrophage-to-faeces reverse cholesterol transport (RCT)^25^,^28^. In this process, apolipoprotein AI-containing nascent HDL act as acceptors for cellular cholesterol from macrophages and other cells from peripheral organs^29^,^30^,^31^,^32^,^33^. Mature HDL particles deliver their cargo to the liver, where cholesterol is selectively taken up by hepatocytes via scavenger receptor B-I (SR-BI) and is processed towards faecal excretion^34^,^35^. The role of plasma HDL-cholesterol levels and their function in RCT with regard to atherosclerosis is controversial^36^. However, recent studies indicate that the cholesterol efflux capacity of HDL rather than total HDL-cholesterol level is associated with cardiovascular outcomes^37^,^38^. The increased cardiovascular risk in patients carrying loss-of-function SR-BI mutations with high plasma HDL-cholesterol underline the relevance of SR-BI-mediated cholesterol flux as an anti-atherogenic mechanism^39^. However, the physiologic mechanisms that increase HDL-mediated cholesterol flux remain unclear, which is of importance for developing targeted HDL therapeutics.

Several lines of evidence indicate a role of thermogenic adipocytes in the metabolism of HDL in humans. Dyslipidemia in patients with obesity and type 2 diabetes, which have lower thermogenic adipocyte activity than healthy subjects^1^,^40^, is characterized by high plasma TG and low plasma HDL-cholesterol concentrations^41^. Interestingly, repeated cryostimulation in humans has been described to lower plasma TG while increasing plasma HDL-cholesterol levels^42^. Likewise, the expression of uncoupling protein-1 (UCP1) in human epicardial fat is associated with lower plasma TG and higher plasma HDL-cholesterol levels^43^. Notably, single-nucleotide polymorphisms in the gene coding for LPL, the master regulator of plasma TG, are linked to increased plasma TG and reduced plasma HDL-cholesterol levels in humans^44^,^45^,^46^. Altogether, based on these findings we hypothesized that the metabolic activity of thermogenic adipocytes is linked to HDL metabolism.

Here we show that thermogenic activation accelerates HDL turnover as well as RCT and leads to characteristic changes of the HDL lipidome in humans and in mice. Although these lipid changes are not associated with altered cholesterol efflux capacity of isolated HDL *ex vivo*, activation of thermogenic adipocytes accelerates plasma cholesterol flux and faecal excretion. Mechanistically, we demonstrate that LPL activation in adipocytes promotes HDL turnover and hepatic clearance of cholesterol in an SR-BI-dependent manner.

## Results

### CL-induced atheroprotection is linked to increased HDL

**Figure 1:**
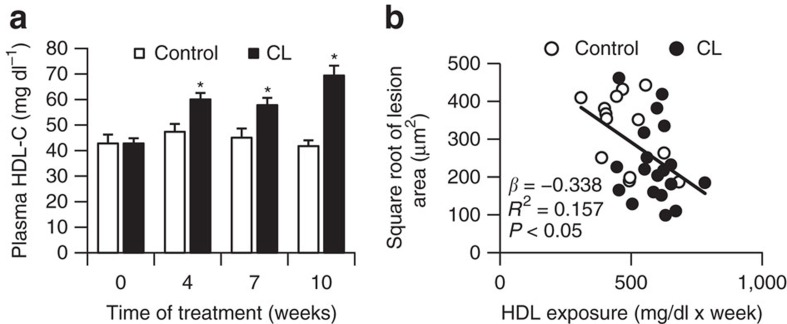
Atheroprotective effects of thermogenic adipocytes are linked to plasma HDL-cholesterol. (**a**) Fasting plasma HDL-cholesterol were measured in Western-type diet-fed female E3L.CETP mice at the indicated time points during treatment with CL316,243 (CL) or vehicle (control; *P<0.05 determined by Student's *t*-test, *n*≥13). (**b**) Correlation of HDL-cholesterol levels in E3L.CETP mice with atherosclerotic plaque size after CL treatment. Values in **a** are means±s.e.m. (*n*≥13 per group). *P<0.05 (**a**,**b**: univariate regression analysis).

Recently, we described that activation of thermogenic adipocytes reduces total cholesterol levels and atherosclerosis by accelerating lipoprotein remnant formation and clearance in female E3L.CETP mice^9^. Interestingly, in addition to lowering of plasma pro-atherogenic non-HDL-cholesterol levels^9^ we found in the current study that chronic CL treatment increased HDL-cholesterol in E3L.CETP mice fed a cholesterol-containing Western-type diet (Fig. 1a). Multiple regression analysis showed that, in addition to plasma non-HDL-cholesterol^9^, plasma HDL-cholesterol was an independent predictor of atherosclerosis development (Fig. 1b). These findings raise the possibility that thermogenic adipocytes modulate HDL composition and/or HDL-mediated cholesterol transport.

### Thermogenic adipocytes modulate HDL-cholesterol in mice

**Figure 2:**
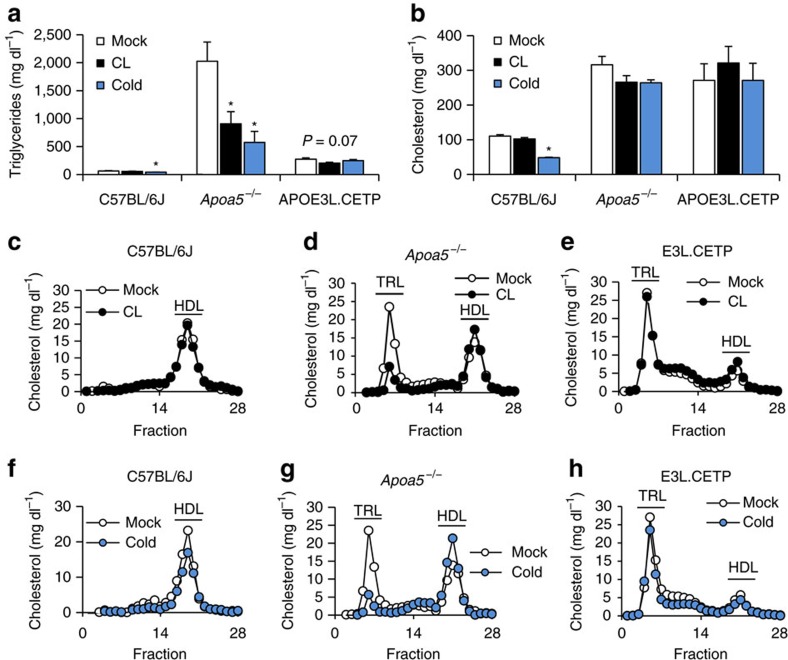
Thermogenic adipocytes modulate TRL and HDL levels in normo- and hyperlipidemic mice. Mice were either mock-treated (Mock), exposed to 4 °C (Cold) or treated with CL316,243 (CL) for 7 days. (**a**) Total plasma TG and (**b**) cholesterol as well as (**c**–**h**) corresponding cholesterol FPLC profiles from plasma of (**c**,**f**) C57BL/6J, (**d**,**g**) *Apoa5*^*−/−*^ and (**e**,**h**) E3L.CETP mice. For FPLC analysis, individual plasma samples were analysed and cholesterol levels were determined n each fraction (*n*=4–5 per group). Values are means±s.e.m. Significance was calculated using unpaired two-tailed Student's *t*-test. *P<0.05, versus mock.

To investigate the effects of thermogenic adipocyte activation on HDL metabolism in mice, we employed a treatment with either cold or CL for 7 days. Both treatments led to BAT activation as well as browning in WAT depots (Supplementary Figs 1 and 2). To study the impact of thermogenic activation on the amount as well as the composition of HDL in the context of different plasma lipid concentrations, we used normolipidemic C57BL/6J wild-type mice, hypertriglyceridemic *Apoa5*^−/−^ mice^47^ as well as hypercholesterolemic E3L.CETP mice. Consistent with previous data^22^, we observed a reduction in total plasma TG, especially in hypertriglyceridemic *Apoa5*^−/−^ mice (Fig. 2a) whereas total plasma cholesterol levels were mostly unaltered (Fig. 2b). In a fast performance liquid chromatography (FPLC) analysis we found that the lipoprotein cholesterol profiles (Fig. 2c,f) and HDL-associated apolipoproteins AI, AV and CIII (Supplementary Fig. 3) of wild-type mice remained largely unchanged. In hypertriglyceridemic *Apoa5*^−/−^ mice both cold and CL treatment induced a shift of cholesterol from the TRL fraction to HDL, increasing the amount of HDL-cholesterol (Fig. 2d,g), whereas in dyslipidemic E3L.CETP mice little change in the cholesterol profile took place (Fig. 2e,h). Thus, a shift of cholesterol from TRL to the HDL fraction was only observed in *Apoa5*^−/−^ mice, which may be explained by a more pronounced reduction of TRL-associated TG compared to the other mouse models (Supplementary Fig. 4).

### Thermogenic adipocytes remodel HDL lipids in mice

**Figure 3:**
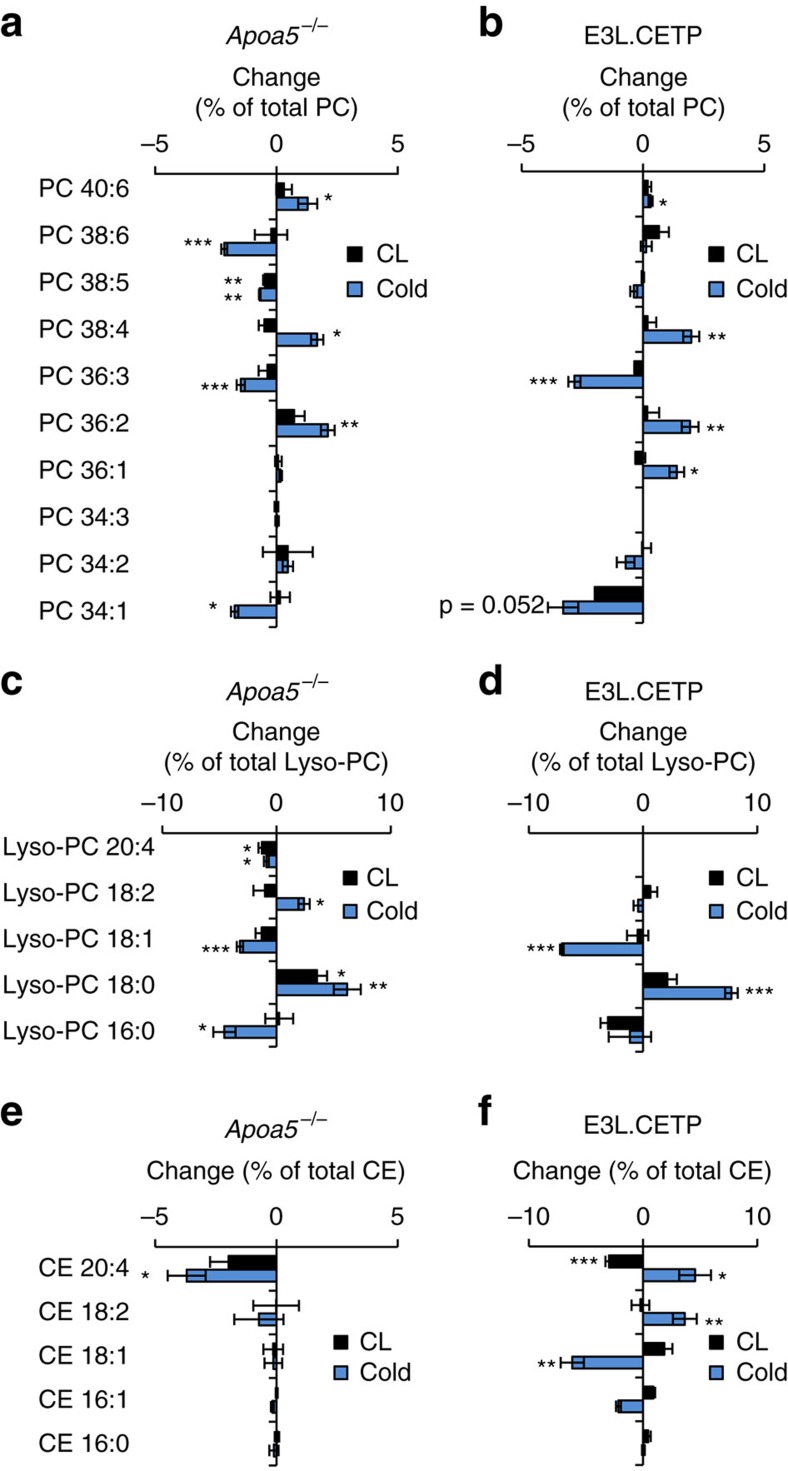
Thermogenic activation causes characteristic HDL lipidome remodelling. Lipidomic analysis of HDL from (**a**,**c**,**e**) *Apoa5*^−/−^ and (**b**,**d**,**f**) E3L.CETP mice after 7 days of CL or cold treatment. Changes in (**a**,**b**) PC, (**c**,**d**) Lyso-PC, (**e**,**f**) CE species relative to mock-treated mice, determined by high-resolution mass spectrometry, are represented as per cent weight changes relative to the whole-lipid class. For example, a 5% change in a PC species translates to, for example, 20% abundance of this lipid to 25% of total PC. Calculated values are mean±s.e.m. (*n*=4-5 per group). *Post hoc* correction for multiple testing was performed using the Benjamini–Hochberg method for the number of lipids shown. *P<0.05, **P<0.01, ***P<0.001, versus mock (Student's *t*-test).

Next to absolute lipoprotein levels, the lipid composition of lipoproteins might be altered after the activation of thermogenic adipocytes. To address this hypothesis, we performed a lipidomic analysis of TRL and HDL particles isolated by FPLC using high-resolution, full scan mass spectrometry with collision-induced dissociation fragmentation. We first studied hyperlipidemic *Apoa5*^−/−^ as they showed the most pronounced shift in lipoprotein pattern in response to thermogenic activation. The lipidomic patterns of HDL exhibited changes after cold exposure and CL treatment (Fig. 3). Thermogenic activation led to remodelling of lipid patterns including phosphatidylcholine (PC; Fig. 3a), lyso-PC (Fig. 3c) as well as cholesteryl ester (CE; Fig. 3e) species. Notably, the relative concentrations of Lyso-PC18:0 and the corresponding PC species PC36:2 were increased, whereas HDL-associated Lyso-PC18:1 and Lyso-PC16:0 as well as the corresponding PC species PC36:3 and PC34:1 were decreased, especially after cold treatment (Fig. 3a,c). In TRL, cold-dependent changes in surface PC patterns were less pronounced but consistent with those in the HDL fraction (Supplementary Fig. 5a). Subsequently, E3L.CETP mice were investigated to address the potential role of the lipid transfer activity mediated by CETP, which is present in humans but not in mice. In E3L.CETP mice, thermogenic activation caused changes in TRL and HDL-associated surface lipids (Fig. 3b,d; Supplementary Fig. 5b), which were very similar to those observed in *Apoa5*^−/−^ mice. Notably, cold-induced changes in CE species were comparable between HDL and TRL in E3L.CETP but not in *Apoa5*^*−/−*^ mice (Fig. 3e,f; Supplementary Fig. 5c,d), indicating exchange of CE species between HDL and TRL mediated by the CETP transgene. Taken together, these data provide evidence for accelerated exchange of PC, Lyso-PC and presumably other (minor) surface lipids between TRL and HDL after thermogenic activation. These findings show that − irrespective of HDL-cholesterol concentrations − the HDL lipid composition is remodelled by cold adaptation and to a similar albeit smaller degree by pharmacologic activation with CL.

### Cold exposure is linked to HDL remodelling in humans

**Figure 4:**
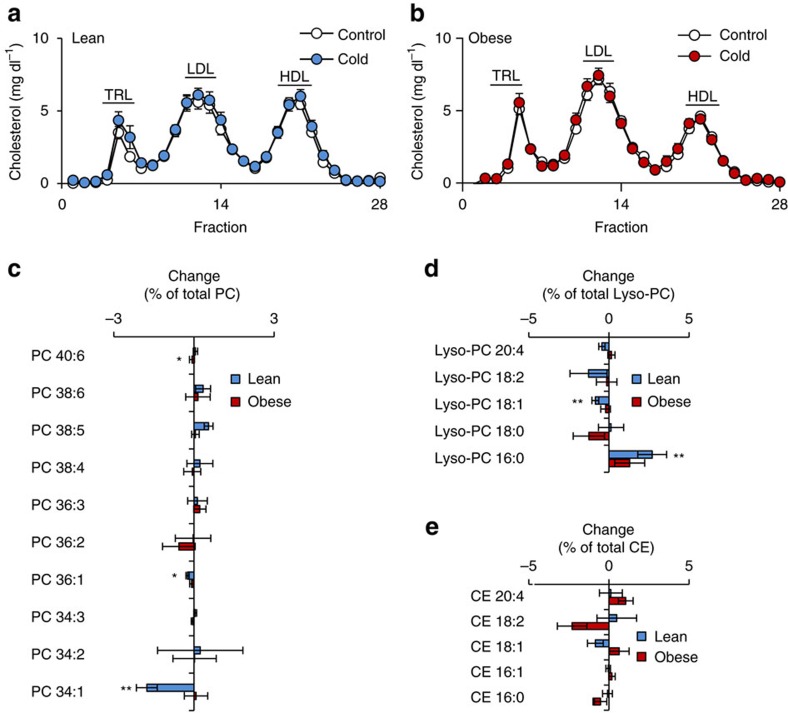
Cold exposure induces HDL lipidome remodelling in humans. Plasma lipoprotein profiles determined by FPLC in (**a**) 9 lean and (**b**) 10 obese humans before and after 2 day-treatment with cold. Changes in (**c**) PC, (**d**) lyso-PC and (**e**) CE species in HDL from cold-treated individuals relative to their baseline levels are represented as per cent weight changes of total lipid class. Calculated values are mean±s.e.m. (*n*=9–10 per group). *Post hoc* correction for multiple testing was performed using the Benjamini–Hochberg method for the number of lipids shown.*P<0.05, **P<0.01, versus mock (Student's *t*-test).

As our results were obtained in preclinical mouse models of hyperlipidemia, we explored how these findings translate into the human situation, in which the activity of thermogenic adipocytes, as determined by ^18^F-fluorodeoxyglucose positron emission tomography–computed tomography (^18^F-FDG-PET-CT), is inversely correlated to BMI and ageing^2^,^4^ but increased by cold exposure^1^,^3^. To address this question, we analysed plasma samples from lean or obese humans that underwent cold exposure for 2 days at 16 °C as previously described^48^. Cold exposure had no significant impact on the lipoprotein profiles of neither lean nor obese probands (Fig. 4a,b and Supplementary Fig. 6a,b). However, when we analysed the lipidomic patterns of isolated lipoprotein fractions, we found that, similar to our observation in mice, HDL-associated PC34:1 and Lyso-PC18:1 were significantly reduced in lean humans by cold exposure (Fig. 4c,d). In addition, Lyso-PC16:0 was significantly increased while no significant changes in CE were observed (Fig. 4e). In obese individuals, however, no changes by cold exposure were observed (Fig. 4c–e), which is in line with the concept that obesity is a state of low thermogenic adipocyte activity. In summary, in both mice and lean humans HDL surface lipids are remodelled in a similar way after cold exposure irrespectively of total plasma HDL-cholesterol concentrations.

### Thermogenic adipocytes promote RCT in mice

**Figure 5:**
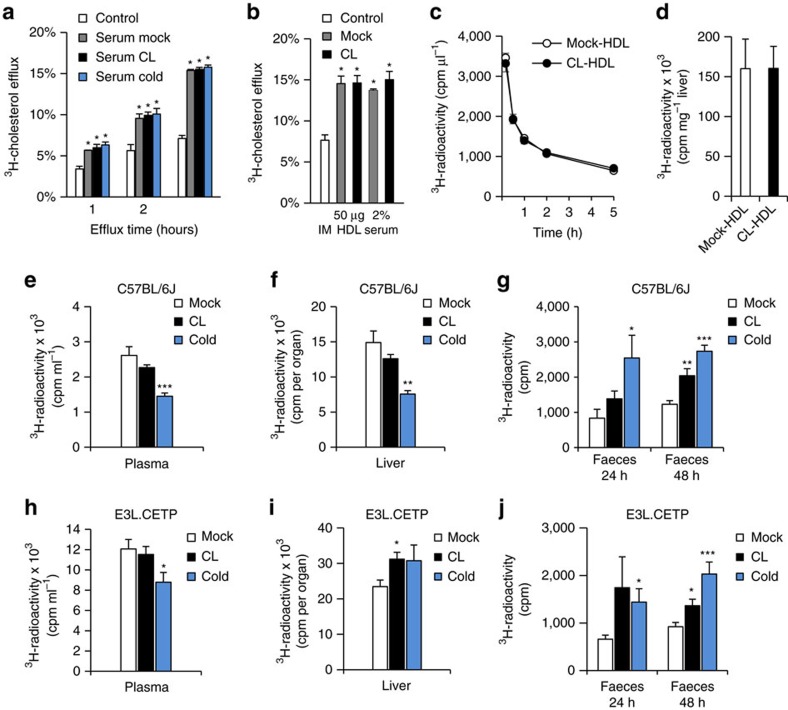
Thermogenic adipocytes promote reverse cholesterol transport without altering cholesterol efflux capacity of HDL. Serum and HDL were prepared from wild-type C57BL/6J mice housed at thermoneutrality (mock), at 4 °C (cold) or treated with CL for 7 days. Peritoneal macrophages were pre-loaded with ^3^H-cholesterol and specific cholesterol efflux was induced (**a**) in the absence (control) or in the presence of 2% serum for the indicated time or (**b**) in the absence (IM=incubation medium) or in the presence of 2% serum or 50 μg ml^−1^ HDL for 4 h at 37 °C. Values are mean±s.e.m. of *n*=3 independent experiments. *P<0.05, versus mock (Student's *t*-test). For metabolic turnover studies, HDL from untreated (mock-HDL) or CL-treated (CL-HDL) wild-type C57BL/6J mice were radiolabelled. After injection of ^125^I-protein shell- and ^3^H-cholesterol oleoyl ether core-radiolabelled HDL, (**c**) plasma clearance and (**d**) liver uptake of ^3^H-cholesterol oleoyl ether were determined. Values are mean±s.e.m. (*n*=7 per group). *In vivo* RCT assay in thermoneutral (mock), CL-treated and cold-exposed (**e**–**g**) C57BL/6J and (**h**–**j**) E3L.CETP mice after the injection of peritoneal macrophages, *ex vivo* loaded with LDL and ^3^H-cholesterol. Radioactivity was determined 48 h after macrophage injection (**e**,**h**) in plasma and (**f**,**i**) in liver as well as (**g**,**j**) 24 h and 48 h after macrophage injection in faeces. Values are mean±s.e.m. (*n*=8–10 per group).*P<0.05, **P<0.01, ***P<0.001, versus mock (Student's *t*-test).

The observed changes in HDL lipid composition might be indicative of altered HDL functions such as cellular cholesterol efflux. To address this hypothesis, we analysed whether HDL isolated from mice after cold adaptation or CL treatment increased cholesterol efflux from primary mouse peritoneal macrophages *ex vivo*. Both serum as well as HDL isolated from control mice promoted cholesterol efflux from macrophages. However, the cholesterol efflux capacity of serum and HDL were not different when HDL were isolated from mice that were cold-adapted or CL-treated (Fig. 5a,b). These data suggest that the lipidomic changes in HDL do not directly alter the capacity of isolated HDL particles to induce cholesterol efflux. Next, we compared the plasma clearance and hepatic uptake of HDL isolated from mock- and CL-treated mice that were radiolabelled *ex vivo* with ^3^H-cholesterylether (a non-hydrolysable marker of the lipid core) and subsequently re-injected into wild-type mice. Both clearance from plasma as well as hepatic uptake of these HDL particles were identical (Fig. 5c,d), indicating that the lipidomic changes in HDL neither directly alter cholesterol efflux nor directly alter the transport capacity of isolated HDL particles. However, this does not rule out that thermogenic adipocytes mediate an enhanced flux of peripherally acquired cholesterol via the HDL compartment towards the liver. To investigate this hypothesis *in vivo*, we performed an RCT assay developed by the Rader laboratory^49^. Briefly, we injected macrophages loaded *ex vivo* with LDL and ^3^H-cholesterol into mice, in which thermogenic adipocytes were activated by cold or CL. In comparison to wild-type mice housed under thermoneutral conditions, cold-adapted mice showed markedly increased ^3^H-cholesterol excretion into faeces, while the concentration of ^3^H-cholesterol in plasma and liver was decreased (Fig. 5e–g). CL treatment led to comparable outcomes including significantly increased faecal excretion of radiolabelled cholesterol. Similarly, we observed increased levels of faecal ^3^H-cholesterol after cold or CL treatment in hyperlipidemic E3L.CETP mice (Fig. 5h–j). Taken together, these data indicate that the activation of thermogenic adipocytes increases cholesterol flux from the periphery to faeces, possibly by accelerated transport via HDL.

### Thermogenic adipocytes accelerate HDL-cholesterol flux in mice

**Figure 6:**
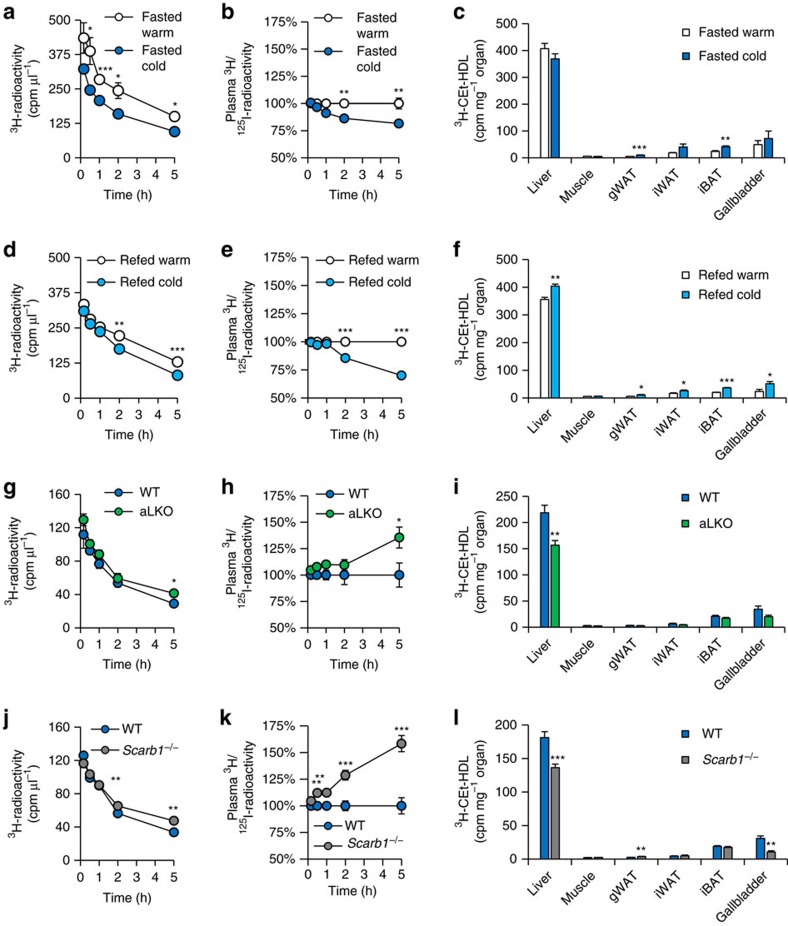
Lipolysis by LPL and hepatic SR-BI increase cold-induced HDL turnover. ^125^I-protein shell- and ^3^H-cholesteryl oleoyl ether (CEt) core-radiolabelled HDL were injected into (**a**–**c**) fasted and (**d**–**f**) re-fed C57BL/6J mice, which had been either cold-exposed for 7 days (cold) or kept under thermoneutral conditions (warm). Double-labelled HDL were also injected into fasted mice cold-adapted for 7 days with (**g**–**i**) adipocyte-specific LPL knockout (aLKO) and (**j**–**l**) *Scarb1*^*−/−*^ mice and wild-type (WT) controls. HDL-derived ^3^H-CEt clearance from plasma was analysed at indicated time points by determining (**a**,**d**,**g**,**j**) total and (**b**,**e**,**h**,**k**) selective clearance. Values for selective clearance are presented as the ratio of ^3^H/^125^I radioactivity and calculated for each time point indicated (an accelerated selective HDL-cholesterol clearance from plasma is indicated by a lower ratio of ^3^H/^125^I while impaired plasma clearance leads to higher ^3^H/^125^I). (**c**,**f**,**i**,**l**) The organ uptake of HDL-^3^H-CEt was measured 5 h after injection of radiolabelled HDL. gWAT, gonadal WAT; iWAT, inguinal WAT; iBAT, interscapular BAT. Values are mean±s.e.m. (*n*=5-7 per group). *P<0.05, **P<0.01, ***P<0.001, versus control (Student's *t*-test).

To unravel whether the observed increase in RCT is dependent on changes in HDL-mediated plasma cholesterol transport to the liver and subsequently to bile, we investigated HDL turnover in cold-adapted and control wild-type mice housed at thermoneutrality. For this purpose, we labelled HDL isolated from wild-type mice with ^3^H-cholesterylether (a marker of cholesterol uptake) and ^125^I-apolipoprotein (a marker of particle uptake)^50^ and injected these radiolabelled particles into controls and mice, in which thermogenic adipocytes were activated. The double-radiolabelling allows the analysis of selective cholesterol plasma clearance^50^. In this set-up, an accelerated selective cholesterol clearance from plasma is indicated by a lower ratio of ^3^H/^125^I while impaired selective plasma clearance results in a higher ^3^H/^125^I ratios. Cold adaptation increased both total and selective cholesterol clearance from plasma without changing the uptake of ^3^H-cholesterylether into the liver of fasted mice (Fig. 6a–c). Next, we performed HDL turnover experiments with re-fed mice, as under this condition LPL activity was increased in adipose tissues (Supplementary Fig. 7). Notably, in this postprandial state with increased plasma lipolysis, both total and selective cholesterol clearance from plasma as well as liver uptake of HDL-derived cholesterylether were increased, accompanied by elevated ^3^H-cholesterylether concentration in the gallbladder (Fig. 6d–f). Interestingly, disposal of ^3^H-cholesterylether (Fig. 6c,f) and to a lesser degree ^125^I-HDL (Supplementary Fig. 8a,b) was also increased in BAT and WAT after cold adaptation, suggesting higher HDL uptake into adipose tissues. The stimulated intravascular lipolytic environment caused by the induction of LPL in thermogenic adipocytes (Supplementary Fig. 7) is likely responsible for the increased HDL turnover and selective cholesterol uptake into liver.

### LPL-mediated lipolysis drives HDL remodelling and turnover in mice

To prove this concept, we analysed the turnover and liver uptake of HDL in cold-exposed mice with adipocyte-specific deletion of LPL (aLKO)^51^. This model shows defective intravascular adipose tissue lipolysis but otherwise thermogenic adipocytes remain intact^51^. Deficiency of adipocyte LPL reduced total and selective plasma cholesterol clearance under conditions of cold adaptation (Fig. 6g,h). Accordingly, we observed reduced uptake of HDL-cholesterol (Fig. 6i) but not of ^125^I-HDL (Supplementary Fig. 8c) into the liver. In line with a prominent role for adipose tissue LPL, thermogenic activation caused lowering of TG levels in wild-type mice but not in aLKO mice (Supplementary Fig. 9a,c,d). Neither total cholesterol nor HDL-cholesterol levels were modulated in this setting (Supplementary Fig. 9b,e,f). However, the mass spectrometry with collision-induced dissociation fragmentation lipidomic analysis confirmed that HDL-TRL remodelling occurred in wild-type controls but was attenuated in aLKO mice (Supplementary Figs 10 and 11). Taken together, these data indicate that LPL-mediated lipolysis by thermogenic adipocytes is a critical driver of HDL remodelling and turnover.

### Cold-induced HDL-cholesterol clearance is mediated by SR-BI in mice

SR-BI (encoded by *Scarb1*) is the major HDL receptor mediating selective cholesterol uptake into liver^34^,^35^. Hepatic levels of SR-BI protein were unchanged in cold-adapted wild-type mice in comparison to controls housed at thermoneutrality (Supplementary Fig. 12), but may still be important for hepatic cholesterol clearance under conditions of activated thermogenic adipocytes. In comparison to wild-type controls, cold-adapted *Scarb1*^*−/−*^ mice showed impaired total and selective cholesterol clearance from plasma as well as decreased uptake of ^3^H-cholesterylether into liver and gallbladder (Fig. 6j–l), while the uptake of ^125^I-HDL was unaltered (Supplementary Fig. 8d). In conclusion, hepatic SR-BI is involved in the accelerated HDL-cholesterol flux induced by thermogenic activation.

## Discussion

Thermogenic brown and beige adipocytes are critical regulators of whole-body energy metabolism and plasma lipoprotein homeostasis^9^,^52^,^53^. While generally the metabolic activity of thermogenic adipocytes is thought to act as a sink for excess calories, in addition our study demonstrates that the activation of thermogenic adipocytes is associated with increased HDL-cholesterol flux from plasma into faeces. To our knowledge this is the first study showing a physiologic condition, in which HDL flux and HDL levels are disconnected. Second, this is the first mechanistic report addressing the relationship of increased metabolism by thermogenic adipocytes and HDL metabolism. We show in genetic loss-of-function models that the increase in HDL turnover was dependent on adipocyte LPL-dependent lipolytic processing and selective HDL-cholesterol disposal in the liver via SR-BI. While our study found substantial changes in the HDL lipidome after stimulation of thermogenic adipocytes, our data indicate that this is not functionally related to increased cholesterol flux through the HDL compartment as observed in the animals. Rather, we propose a mechanism where lipolysis-driven transfer of TRL-derived surface remnants to HDL, as proposed by Eisenberg *et al*. based on *in vitro* experiments^54^,^55^, accelerates the metabolic flux of HDL-cholesterol to the liver. Supporting this hypothesis, changes in PC species induced by cold adaptation were similar in HDL and TRL. Interestingly, the exchange of CE species between HDL and TRL particles was only observed in E3L.CETP but not *Apoa5*^*−/−*^ mice, confirming that CETP activity promotes the transfer of core lipids also in the context of thermogenic adipocyte stimulation. In the future, it will be important to investigate the mechanistic details of lipid exchanges between lipoprotein particles regarding lipid classes, lipid species, directionality, enzymes and transfer proteins under conditions of thermogenic activation. In this context, it will be also important to investigate how our results in normo- and hyperlipidemic mouse models here can be translated into humans with different types of dyslipidemias. For instance, the effect of thermogenic adipocytes on HDL is likely masked in hypertriglyceridemic patients without functional LPL activity.

Thermogenic activation by cold exposure is accompanied by a robust increase in food intake to maintain systemic energy homeostasis. The resulting increased intake of cholesterol or related sterols requires adaptive mechanisms to avoid a systemic sterol overload. This is particularly important for BAT, which, when active, takes up whole TRL lipoprotein particles including cholesterol^53^,^56^. One mechanism counteracting cholesterol accumulation in thermogenic adipocytes might involve direct cholesterol efflux from adipocytes to HDL. Adipocyte ABCA1 has been demonstrated to contribute to HDL maturation both *in vivo*^57^ and *in vitro*^58^. In addition, genetic ablation of ABCG1 results in massive lipid accumulation in multiple tissues after feeding a cholesterol-containing diet, offering the possibility that ABCG1 may also play a critical role in WAT or BAT cholesterol homeostasis^59^. Experiments using mice lacking ABCA1 or ABCG1 specifically in thermogenic adipocytes will be needed to show whether the high lipolytic activity of brown and beige adipocytes not only increases intravascular HDL remodelling and turnover as shown in this study, but also enhances cholesterol efflux from brown and beige adipocytes^60^. On the other hand, intestinal cholesterol handling could also be altered in the setting of cold exposure, which might contribute to the stimulated RCT. In line, decreased intestinal re-absorption of cholesterol was previously found as the cause of reduced RCT in a mouse model of restraint stress^61^.

We found that the characteristic changes in the HDL lipidome were basically also observed in lean humans, but, interestingly, not in obese probands. It remains to be proven, however, whether the decrease of human brown fat activity that is observed with ageing, obesity and insulin resistance^2^,^5^,^62^,^63^ is causally linked to a general deterioration of plasma lipids homeostasis and increased risk of cardiovascular disease (CVD)^64^. On the other hand, the linkage of TG turnover and HDL-cholesterol flux under conditions of increased energy expenditure exemplified here by the stimulation of thermogenic adipocytes might also be causally involved in the relationship between sedentary lifestyles and cardiovascular risk^65^. Also, as cold adaptation involves a certain contribution of shivering thermogenesis, it might explain why we found that cold adaptation has more pronounced effects on HDL metabolism than CL treatment alone, indicating a possible contribution of muscle-mediated lipolysis during cold. Furthermore, as we used Fabp4-Cre to delete LPL from adipocytes, it is possible that other cell types like macrophages play an additional role in the processing of TRL and HDL under cold conditions. In light of recent studies demonstrating the therapeutic potential of BAT activation in humans^10^,^12^,^13^, it is tempting to speculate that stimulating thermogenic adipocyte activity may protect from cardiovascular disease not only by decreasing remnant lipoproteins levels^9^ but also by stimulating HDL-cholesterol flux from atherogenic lesions to the liver for ultimate cholesterol excretion.

## Methods

### Experimental animals and diets

All animal experiments were approved by the Animal Welfare Officers of University Medical Center Hamburg-Eppendorf (UKE) and Behörde für Gesundheit und Verbraucherschutz Hamburg as well as the Institutional Ethical Committee on Animal Care and Experimentation of the Leiden University Medical Center. Mice were bred in the animal facilities at 22 °C with a day and night cycle of 12 h. We used male age-matched (12–18 weeks): C57BL/6J wild-type mice were received from Charles River Germany; *Fabp4-Cre+* mice from Jackson Laboratories (Bar Harbor, ME; # 005069); *Lpl*-floxed mice from Jackson Laboratories (Bar Harbor, ME; # 006503), *Scarb1*^–/–^ from Jackson Laboratories (Bar Harbor, ME; # 003379). These mice were obtained on a C57BL/6J background and were bred at the UKE animal facilities. Breeder pairs of *Apoa5*^–/–^ mice were kindly provided by Dr Len Pennacchio (Lawrence Berkeley National Laboratory) and backcrossed to the FVB background for more than 10 generations. *E3L* female mice (line 2; generated at Leiden University Medical Center) crossbred with mice expressing human CE transfer protein (received from Jackson Laboratories, Bar Harbor, ME; # 003904) to generate *E3L.CETP* on C57BL/6J background^66^. Animals had *ad libitum* access to food and water. For the experiments, mice were randomized based on their body weights and were housed in single cages and fed a standard chow diet (Lasvendi), unless indicated otherwise. Cold exposure was performed by housing mice at 4 °C for 1 week, whereas control mice were housed at thermoneutrality. The β_3_-adrenergic agonist CL316,243 (Tocris, 0.2 mg ml^−1^ in 0.9 w/v % NaCl) was administered by subcutaneous injection (1 μg per g body weight per day) for 7 days, mock-treated control mice received 0.9 w/v % NaCl correspondingly.

If not stated otherwise, tissue and blood collections were performed after 4 h fasting initiated between 8:00 and 9:00 am. Mice were anaesthetized with a lethal dose (15 μl g^−1^ mouse body weight) of a mix containing Ketamin (25 mg ml^−1^)/Xylazin (0.2%) in 0.9% NaCl. Blood was withdrawn transcardially with syringes containing 0.5 M EDTA for plasma preparation and animals were perfused with 5 ml ice-cold PBS containing 10 U ml^−1^ heparin. Organs were harvested and immediately conserved either in TRIzol reagent (Invitrogen), phosphate-buffered 3.7% formaldehyde or snap-frozen in liquid N_2_ and stored at −80 °C for further processing. For practical reasons, investigators were not blinded as to the group allocation.

### Plasma parameters

Plasma TG and cholesterol were measured using commercial kits (Roche) that were adapted to 96-well microtiter plates. HDL-cholesterol levels were measured after precipitating apoB-containing lipoproteins from plasma by addition of 20% polyethylene glycol in 200 mM glycine buffer with pH 10. For lipoprotein profiling, 200 μl of plasma was separated by FPLC using a Superose 6 10/300 GL column (GE Healthcare) with a flow rate of 0.5 ml min^−1^. 28 fractions (volume of factions 0.5 ml) were collected. In each fraction, TG as well as cholesterol levels were determined. For further analysis, FPLC fractions 4-6 (TRLs) and 17-20 (HDL) were pooled (350 μl per well) and stored at −80 °C immediately after FPLC for further processing.

### Lipid extraction and high-resolution lipidomics

FPLC fractions were spiked with several internal standards (see list Supplementary Table 1; obtained from Sigma-Aldrich or Avanti Polar Lipids) before lipid extraction by the method of Bligh and Dyer^67^, respectively. The lipid extraction of FPLC fractions was performed with 320 μl of the pooled FPLC fractions (TRL, HDL; see above). After addition of 1,000 μl methanol, 50 μl internal standard (diluted 1:10) and 700 μl chloroform the tube is stirred for 30 s on a Vortex-shaker. 1,100 μl chloroform and 900 μl MS-grade-water was added and the solution was again stirred for 30 s on a Vortex-shaker. Samples were centrifuged at 4 °C for 15 min at 3,000 g. 1,600 μl of the lower organic phase was transferred to a new glass tube and the solvent was evaporated to dryness by vacuum centrifugation using a ZentriVac. The lipid extracts were resuspended in 80 μl of eluent B and transferred to glass vials. Lipidomic analyses were conducted on a Dionex3000 UPLC (Column: Kinetex C18, 150 × 2.1 mm; 1.7 μm (Phenomenex)) coupled to an ESI-UHR-Q-TOF mass spectromereeter (maXis3G, Bruker Daltonik). Data w acquired using high-resolution, full scan MS and collisional induced dissociation fragmentation. For chromatography the following gradient with a flow rate of 300 μl min^−1^ was performed: 0 min 80% B, in 2 min to 87% B, 6 min isocratic, in 2 min to 95% B, in 10 min to 99% B, 1 min isocratic, in 1 min to 80% B, 3 min isocratic. Eluent A consisted of ultra-pure water enriched with 5 mM NH_4_Ac, eluent B of MeOH/IPA, 4/6 (vol/vol) enriched with 5 mM NH_4_Ac. Column temperature was maintained at 55 °C. The mass spectrometer was operated in positive ionization mode: A capillary voltage of 4.5 kV and an end plate offset voltage of −500 V were applied. The inlet LC flow was nebulized using nitrogen gas (2 bar), the dry temperature was kept at 190 °C. Data were acquired over the mass range of 100–1,000 Da for both MS and MS/MS modes. Nitrogen was used for collisional induced dissociation with collision energies between 14 and 35. Obtained spectra were externally calibrated on ESI-L tuning mix (Agilent Technologies) running via syringe pump at 20 μl h^−1^. Further internal calibration was performed for each sample by using the lockmass hexakis (1H, 1H, 2H-perfluoroethoxy) phosphazene (Apollo Scientific Limited). UPLC-MS data were processed using the manufacturer software (DataAnalysis 4.0 and TargetAnalysis 1.3). Only peak areas of individual lipid species that were within the range of external calibration curves were calculated by comparing the individual peak areas with those of corresponding internal standards for determining the final concentrations. Lipid species were identified by means of standard substances, MS/MS-spectra (see list of fragment sizes in Supplementary Table 2) and the LIPID MAPS database (http://www.lipidmaps.org/tools/index.html). We detected 7-15 CEs, 7-15 LysoPCs and 18-30 PCs. However, Figs 3 and 4 show the ten or five most consistently detected species for the depicted lipid classes representing the highest common denominator of all analyses.

### Human HDL

All participants signed an informed consent for the study protocol, which was approved by the institutional review board of Maastricht University Medical Center. Blood samples were obtained from nine male lean (BMI 20.8–24.8 kg m^−2^) and 10 male obese (BMI 28.6–40.8 kg m^−2^) subjects after they stayed for 36 h at a neutral temperature (22 °C, control) and after 48 h exposure to mild cold (16 °C, cold) as described previously^48^. In brief, subjects stayed in a respiration chamber for 84 h with standardized clothing. The first 36 h the chamber temperature was set to 22 °C (baseline); the following 48 h chamber temperature was set to 16 °C (mild cold exposure). The temperature of the mild cold situation (16 °C) has been validated earlier with similar clothing to be slightly above the shivering threshold. Blood samples were taken in the fasted state under baseline conditions and 48 h after mild cold exposure. EDTA-plasma was obtained by centrifugation, frozen in liquid nitrogen and stored at −80 °C until further analysis. Individual HDL samples were isolated by FPLC using a Superose 6 column (GE Healthcare Life Sciences) from lean and obese subjects before and after cold exposure. Lipid species were determined by UPLC-ESI-UHR-TOF as described above.

### Gene expression analysis

Total RNA was isolated from tissue samples using NucleoSpin RNA II kit (Macherey & Nagel). Synthesis of complementary DNA was performed using SuperScript III Reverse Transcriptase (Invitrogen). Quantitative real-time PCR reactions for indicated genes were conducted on a 7900HT sequence detection system (Applied Biosystems) using TaqManAssay-on-Demand primer sets (Applied Biosystems, *Ucp1*: Mm00494069_m1, Ppargc1a: Mm00447183_m1) supplied by Applied Biosystems and selected to recognize RefSeq sequences and a maximum of Genbank ESTs. Gene of interest cycle thresholds (Cts) were normalized to *TATA-box binding protein* (*Tbp*) house keeper levels by the ΔΔCt method and displayed as relative copies per *Tbp* or relative expression normalized to experimental control groups.

### Western blot

Frozen liver samples were prepared for SDS–PAGE using a radioimmunoprecipitation assay buffer supplemented with protease inhibitors (Roche). Protein samples (15 μg per lane) were separated on a 10% Bis-Tris (pH 6.6) polyacrylamide gel using NuPAGE MES SDS Running Buffer under reducing conditions (Invitrogen). After the transfer to nitrocellulose membranes, blots were blocked for 2 h in PanReac Blocking buffer (AppliChem) and incubated for 1 h at room temperature with the primary antibody against SR-B1 (NB400-101; Novus Biologicals) and against β-actin (A2228; Sigma). Membranes were incubated for 90 min in secondary horseradish peroxidase conjugated antibodies (goat anti-rabbit, Jackson Immunoresearch, 1:5,000). Detection of protein bands was performed using a luminol/para-hydroxycoumarinic acid-based chemiluminescence substrate.

### HDL turnover assay

For HDL preparation, C57BL/6J WT mice were fasted 4 h before blood withdrawal. After isolation of HDL (*d*=1.063–1.21 g ml^−1^) by sequential ultracentrifugation^68^, purified HDL were double-labelled with ^125^I-tyramine cellobiose (^125^I-TC) in the apolipoprotein moiety and with ^3^H-Cholesteryl oleoyl ether (CEt) in the lipoprotein core^35^. Using human plasma CETP, ^3^H-CEt was introduced into ^125^I-TC-HDL by exchange from donor liposomal particles, which contained ^3^H-Cet. The final ^125^I-TC-/^3^H-Cet-HDL particles were dialyzed against PBS (pH 7.4, 4 °C) containing 1 mM EDTA.

For analysis of plasma decay and organ uptake of radiolabelled HDL, mice were fasted for 4 h before tracer injection. After injection of ^125^I-TC- / ^3^H-CEt-HDL (30 μg HDL protein per mouse; ca. 39 kBq ^125^I-TC and 33 kBq ^3^H-CEt, respectively) via the tail vein, blood samples were collected at given times after injection: 10 and 30 min; 1, 2 and 5 h. Plasma aliquots and tissues were directly assayed for ^125^I radioactivity whereas ^3^H-radioactivity was analysed by scintillation counting after lipid extraction using the method of Dole^69^.

### *In vivo* reverse cholesterol transport assay

*In vivo* RCT was assayed by a method developed by Dan Rader and colleagues^49^. For the preparation of peritoneal macrophages, *Ldlr*^–/–^ mice were injected with 2 ml thioglycollate 4 days before isolation by peritoneal lavage with warm DMEM. Cells were plated and after 4 h radiolabelled *ex vivo* with tracer ^3^H-cholesterol (100 kBq corresponding to 1.5 × 10^6^ cells per mouse) and loaded with cholesterol by overnight incubation with acetylated LDL (20 μg ml^−1^). To avoid shear stress we used 0.55 25 mm 24 G × 1′′ Gr. 17 needles (Braun) for the intraperitoneal injection of 1 × 10^6^ radiolabelled macrophages. During the 48 h experiment, mice were kept in special cages that prevented the mice from tampering with the faeces. Faeces, tissue and blood collections were performed 48 h after macrophage injection. ^3^H-radioactivity of plasma, faeces and organs was measured as described above.

### *In vitro* cholesterol efflux assay

Peritoneal macrophages and HDL from control, CL-treated and cold-exposed were isolated as described above. Cells were incubated overnight at 37 °C with ^3^H-cholesterol (37 kBq ml^−1^) and 20 μg ml^−1^ LDL in DMEM containing 0.1% BSA. Then, cells were washed using pre-warmed DMEM and surface-bound LDL was removed using 100 U heparin diluted in DMEM. Cells were incubated for additional 240 min at 37 °C in DMEM+0.1% BSA to allow unspecific cholesterol efflux. Then, cells were washed using pre-warmed DMEM and specific cholesterol efflux was induced in the absence or in the presence of 2% serum or 50 μg/ml HDL for 60-240 min at 37 °C. Media were harvested and cells were lysed in 0.1 N NaOH, radioactivity was determined by scintillation counting and ^3^H-cholesterol efflux was calculated in %.

### Histology

Anaesthetized mice were perfused with PBS containing 2% formaldehyde. Adipose tissues were embedded in paraffin. Haematoxylin and eosin staining was performed using standard protocols as described^70^.

### Statistics

Data are expressed as mean±s.e.m. Student's *t*-test, one- or two-way ANOVA were used for comparison between groups. The estimated variation within each group of an experiment was similar. No statistical method was used to predetermine sample size. For comparison of lipidomic data, *post hoc* correction for multiple testing was performed using the Benjamini–Hochberg method for the number of lipids shown in the respective figures. Univariate regression analyses were performed to test for significant correlations. Square root of the lesion area was taken to linearize the relationship with the plasma HDL-cholesterol exposure in *E3L.CETP* mice. GraphPad Prism 5.0 or SPSS 20.0 were used for statistical calculations with statistically significant differences denoted as *P<0.05.

### Data availability

The datasets generated during and/or analysed during the current study are available from the corresponding authors upon reasonable request.

## Additional information

**How to cite this article:** Bartelt, A. *et al*. Thermogenic adipocytes promote HDL turnover and reverse cholesterol transport. *Nat. Commun.* **8,** 15010 doi: 10.1038/ncomms15010 (2017).

**Publisher's note:** Springer Nature remains neutral with regard to jurisdictional claims in published maps and institutional affiliations.

## Supplementary Material

Supplementary Information Supplementary Figures, Supplementary Tables.
