# Supplementary material for: Thermogenic adipocytes promote HDL turnover and reverse cholesterol transport
Source: Nat Commun. 2017 Apr 19;8:15010. doi: 10.1038/ncomms15010 (PMC5399294; doi:10.1038/ncomms15010)
Supplement: Supplementary Information — Supplementary Figures, Supplementary Tables. [file ncomms15010-s1.pdf]

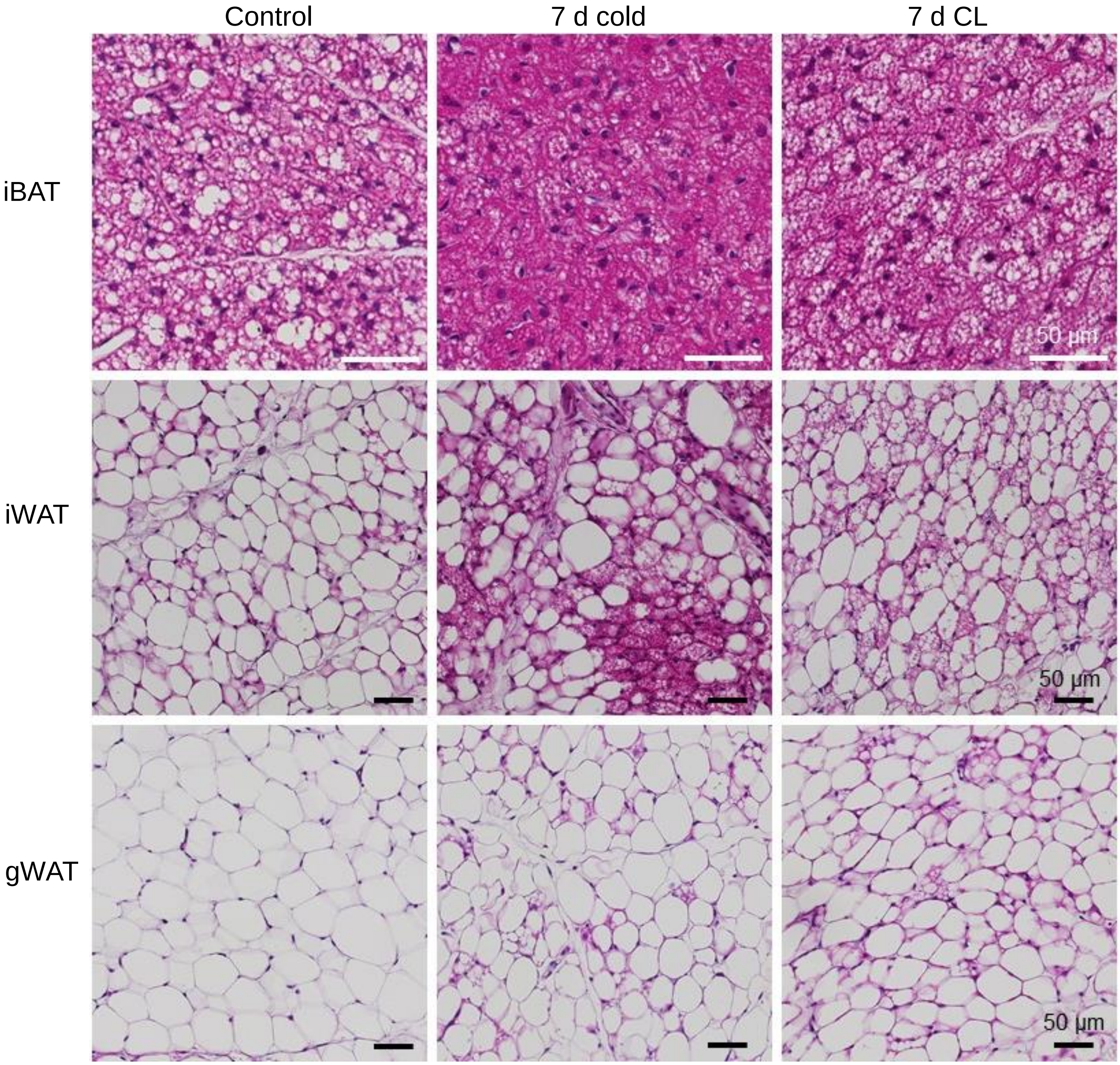

Supplementary Figure 1. Histology of adipose tissues after cold or β3-adrenergic receptor stimulation. C57BL/6J wild-type mice were housed at 4 °C or injected daily with β3-adrenergic receptor agonist CL316,243 (CL) for 7 days. Interscapular brown adipose tissue (iBAT), inguinal white adipose tissue (iWAT) and gonadal white adipose tissue (gWAT) were dissected, fixed in paraformaldehyde and paraffin sections were HE-stained. Bar = 50 µm.

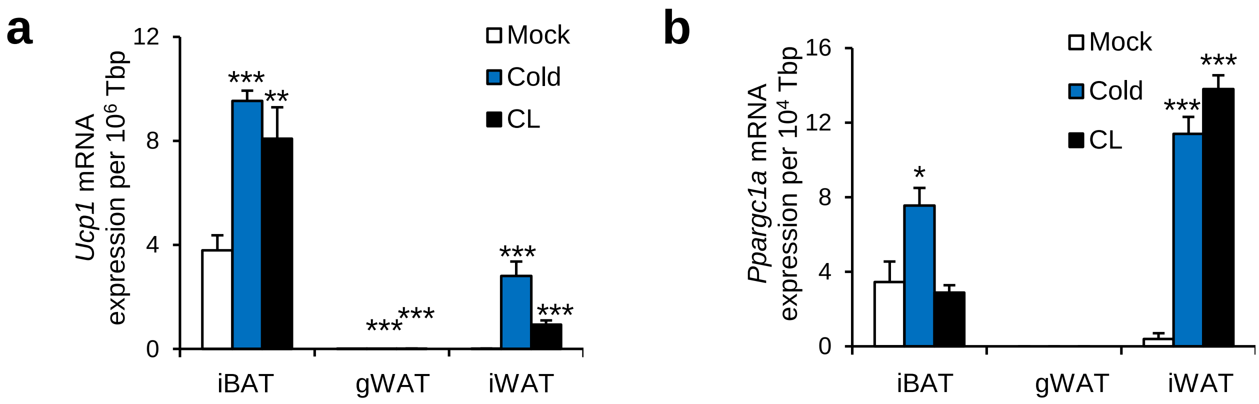

Supplementary Figure 2. BAT activation and WAT browning indicated by increased *Ucp1* and *Ppargc1a* expression. C57BL6/J wild-type mice were exposed to 4 °C or treated with CL316,243 (CL) for 7 days. (a) *Ucp1* and (b) *Ppargc1a* gene expression were determined in interscapular brown adipose tissue (iBAT), inguinal white adipose tissue (iWAT) and gonadal white adipose tissue (gWAT). Values are means ± s.e.m. (n=5 per group). Significance was calculated using unpaired two-tailed Student's t-test. **P*≤0.05, ***P*≤0.01, ****P*≤0.001, versus mock.

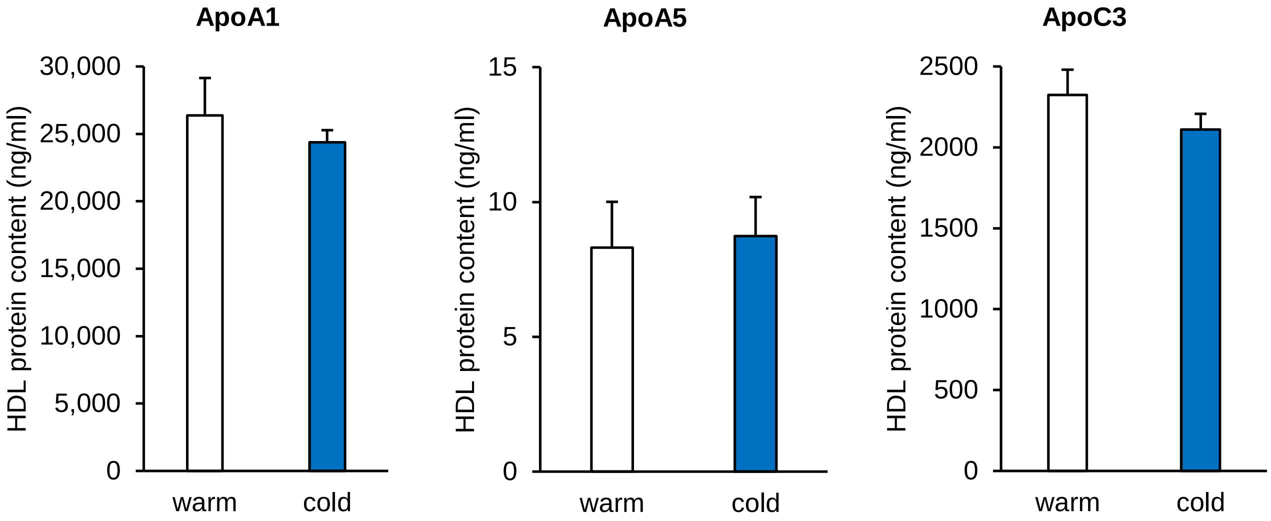

Supplementary Figure 3. Apolipoprotein levels in isolated HDL are unchanged upon cold exposure. C57Bl/6J wild-type mice were housed under thermoneutral (warm) or cold conditions for 7 days. HDL were isolated by FPLC from plasma samples of fasted mice (n=4 per group) and apolipoprotein content of murine ApoA1, ApoA5 and ApoC3 associated with HDL were determined by commercial ELISAs (Cloud Clone Corp, USA). Values are means ± s.e.m.

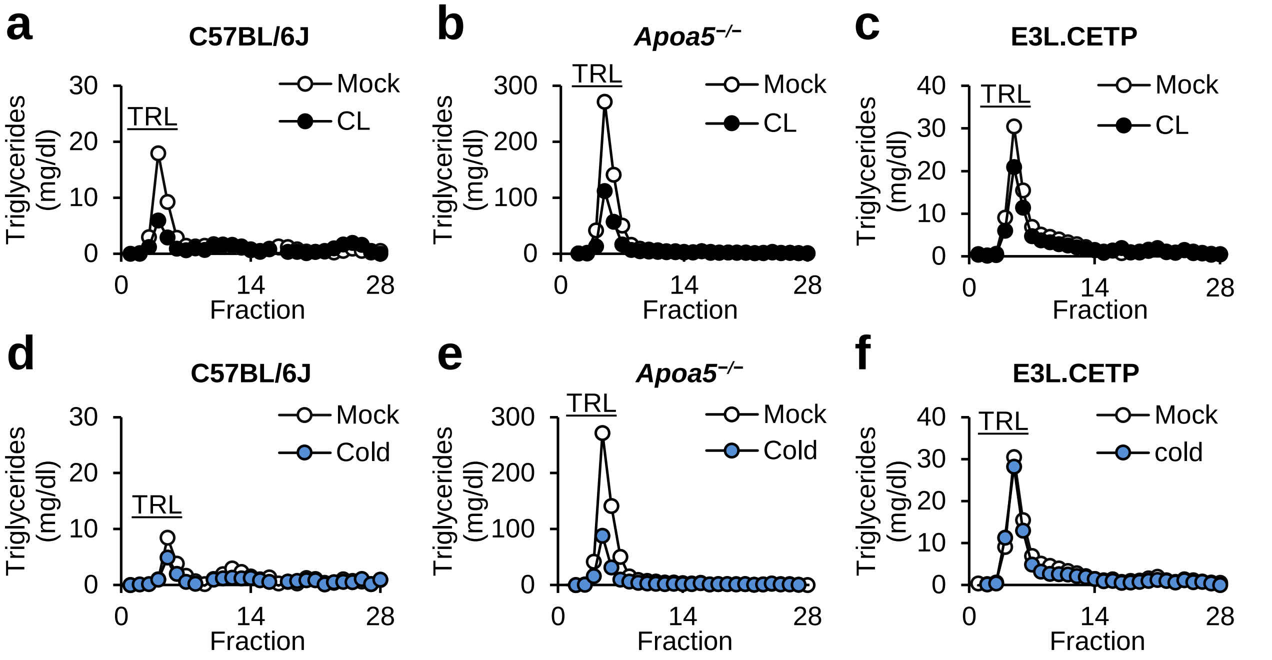

Supplementary Figure 4. Plasma lipoprotein triglyceride profiles determined by FPLC. C57BL/6J wild-type mice, *Apoa5*$^{-/-}$ mice and E3L.CETP mice were either mock-treated, exposed to cold or treated with CL316,243 (CL) for 7 days. Individual plasma samples were separated by FPLC and triglyceride levels were determined in each fraction. Values are means (n=4-5 per group).

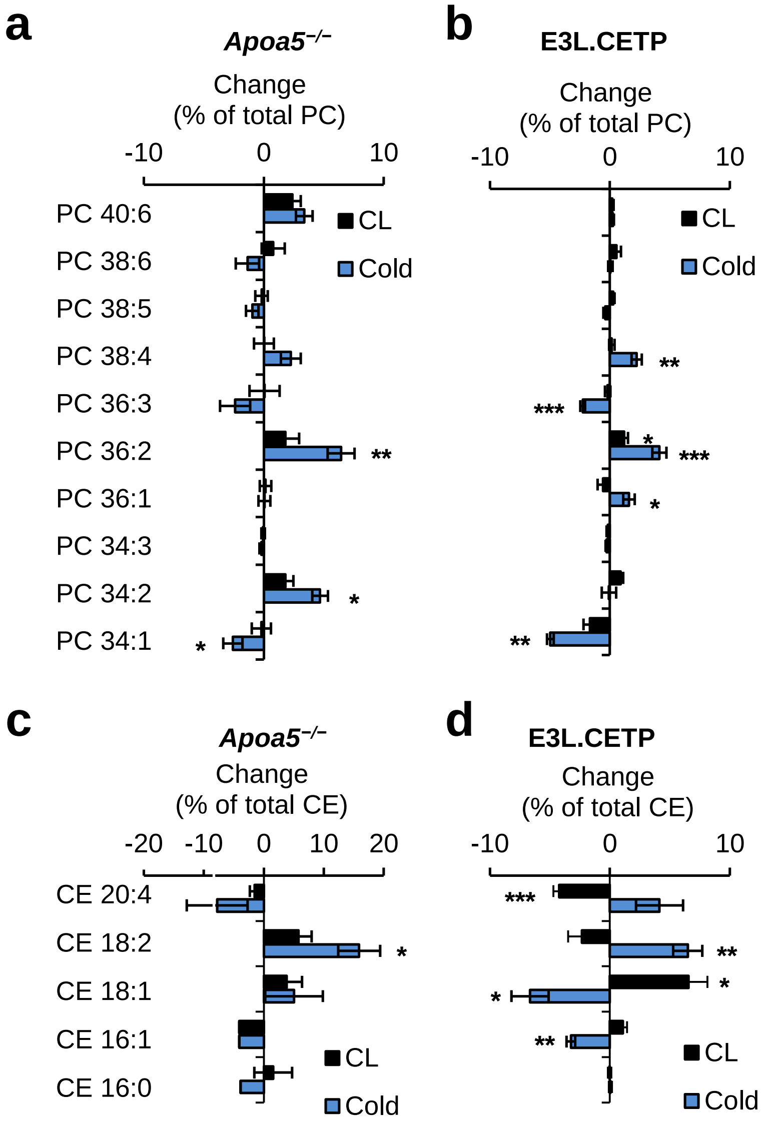

Supplementary Figure 5. Lipidomic changes of TRL from *Apoa5*$^{-/-}$ and E3L.CETP mice after thermogenic activation. *Apoa5*$^{-/-}$ mice and E3L.CETP mice were either mock-treated, exposed to cold or treated with CL316,243 (CL) for 7 days. Lipids of TRL from (**a**,**c**) *Apoa5*$^{-/-}$ and (**b**,**d**) E3L.CETP mice were determined by MS-CID (Lyso-PCs were undetectable). Changes of (**a**,**b**) PC and (**c**,**d**) CE species are presented relative to mock-treated mice as change of % (weight) of total lipid class. For example, if a PC species has an abundance of 20% of total PC and increases to 25% of total PC after thermogenic stimulation, this is shown as +5%. Calculated values are mean ± s.e.m. (n= 4-5 per group). * *P*<0.05, ** *P*<0.01, *** *P*<0.001, versus mock (Student's t-test).

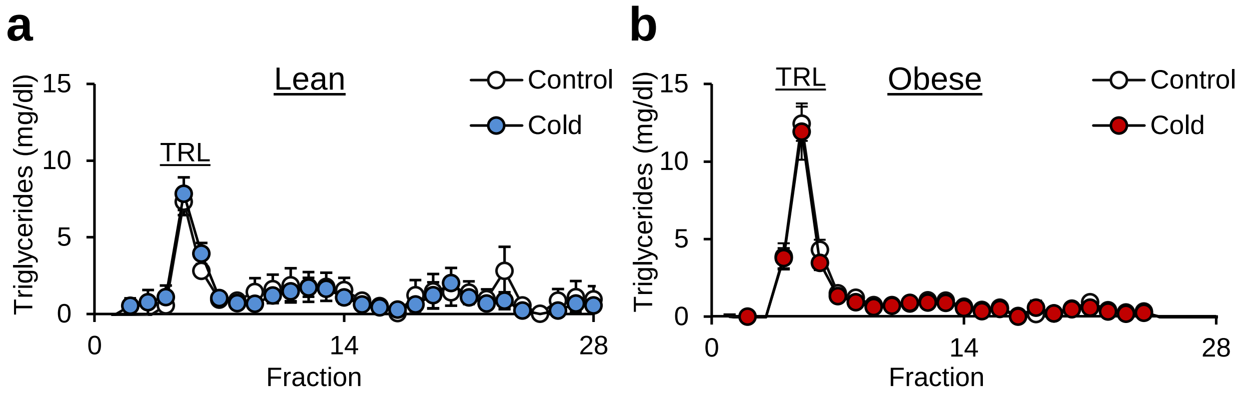

Supplementary Figure 6. Triglyceride lipoprotein profiles of plasma samples from lean and obese humans before and after cold activation. Plasma lipoprotein profiles from (**a**) lean and (**b**) obese subjects untreated (control) or cold exposed (cold) were determined by FPLC. Values are mean ± s.e.m. (n= 10 per group).

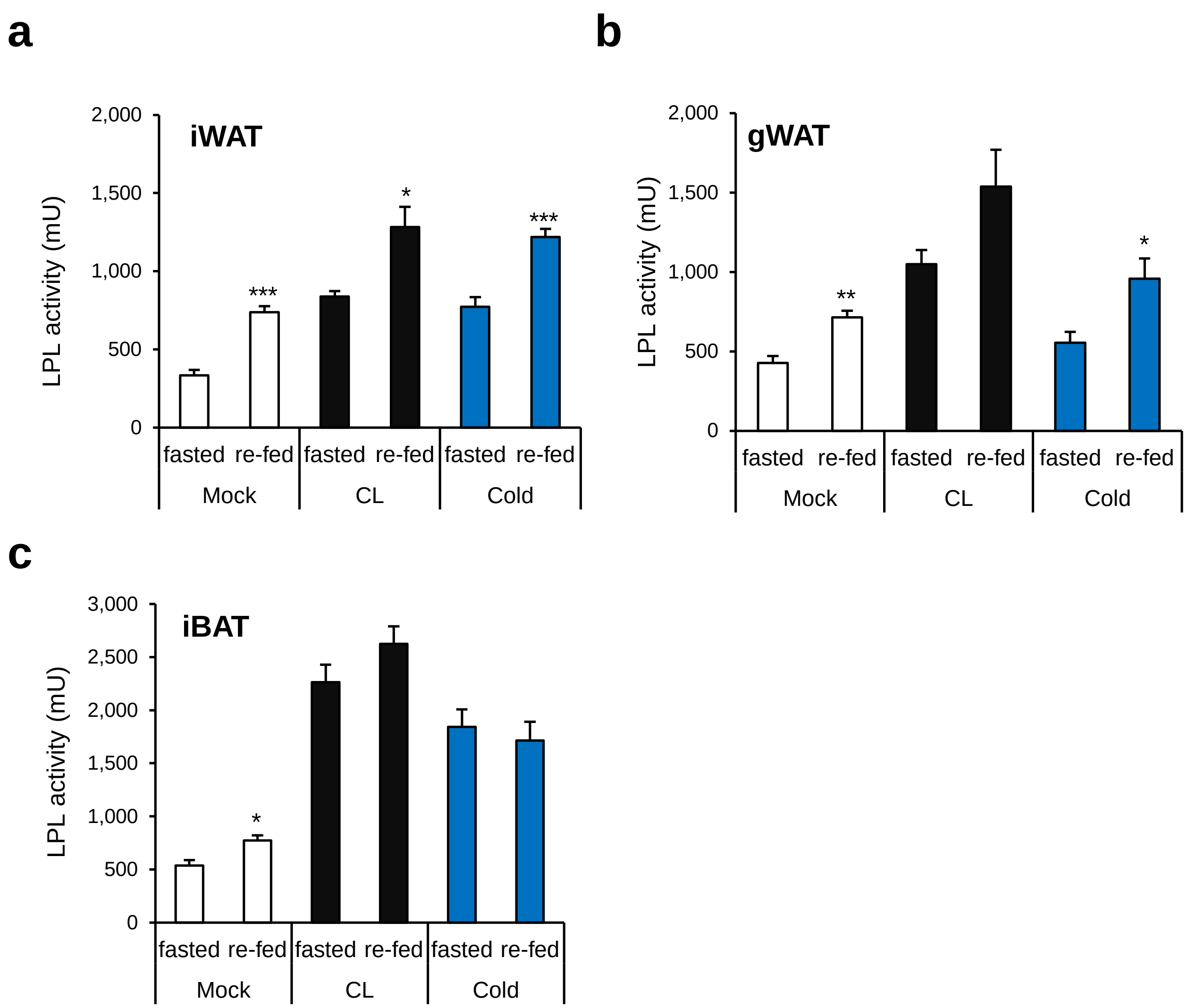

Supplementary Figure 7. LPL activity in adipose tissues in fasted and refed mice after cold or β3-adrenergic receptor stimulation. Mice were exposed to 4 °C or treated with 1 mg/kg CL316,243 (CL) for 7 days. Tissues were harvested after a 4 h fasting period or after 4 h fasting and subsequent 3 h re-feeding. LPL activity was determined in tissue homogenates of (**a**) iWAT, (**b**) gWAT and (**c**) iBAT using radioactive substrate as described (1). Values are means ± s.e.m. (n=5-6 per group). Significance was calculated using unpaired two-tailed Student's t-test. **P*≤0.05, ***P*≤0.01, ****P*≤0.001, fasted versus re-fed.

1.) Dijk W, Heine M, Vergnes L, Boon MR, Schaart G, Hesselink MK, Reue K, van Marken Lichtenbelt WD, Olivecrona G, Rensen PC, Heeren J, Kersten S. ANGPTL4 mediates shuttling of lipid fuel to brown adipose tissue during sustained cold exposure. Elife. 2015; 4. pii: e08428.

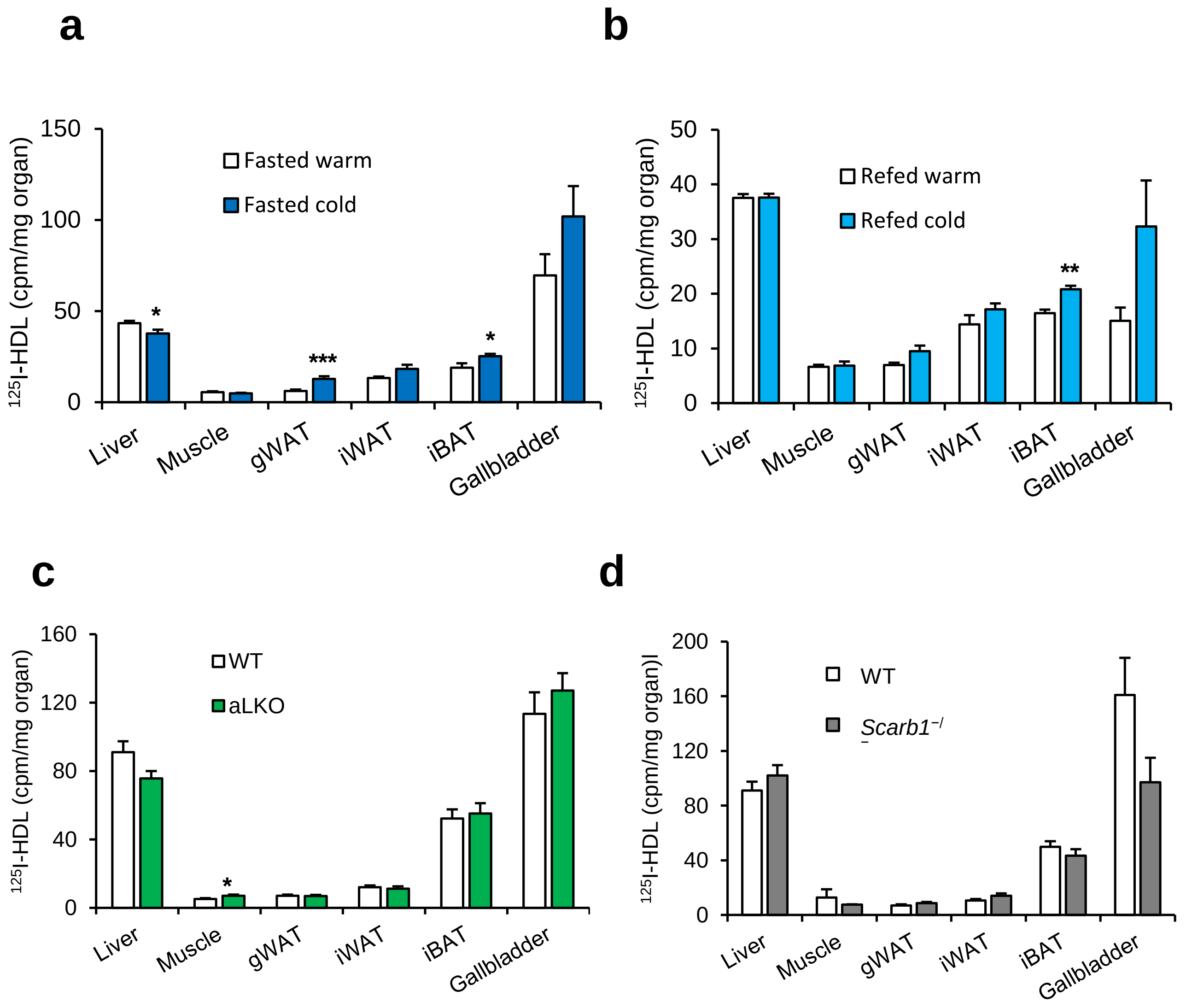

Supplementary Figure 8. Cold-induced accelerated HDL-turnover is dependent on adipocyte LPL and SR-BI. ^{125}I-protein shell- and ^{3}H-cholesterolether core-labeled HDL were injected into fasted (**a**) and refed (**b**) C57BL/6J wild-type mice (WT) which had been either cold-exposed for 7 days (cold) or kept under control conditions at 28°C (warm). Double-labeled HDL were also injected into fasted mice with adipocyte-specific LPL knockout (aLKO) (**c**) and *Scarb1*$^{-/-}$ mice (**d**) and WT controls, all cold-treated for 7 days. ^{125}I-HDL organ uptake was measured 5 h after injection. Values are mean ± s.e.m. (n= 5-7 per group). *P<0.05, ** P<0.01, P<0.001 (Student's t-test).

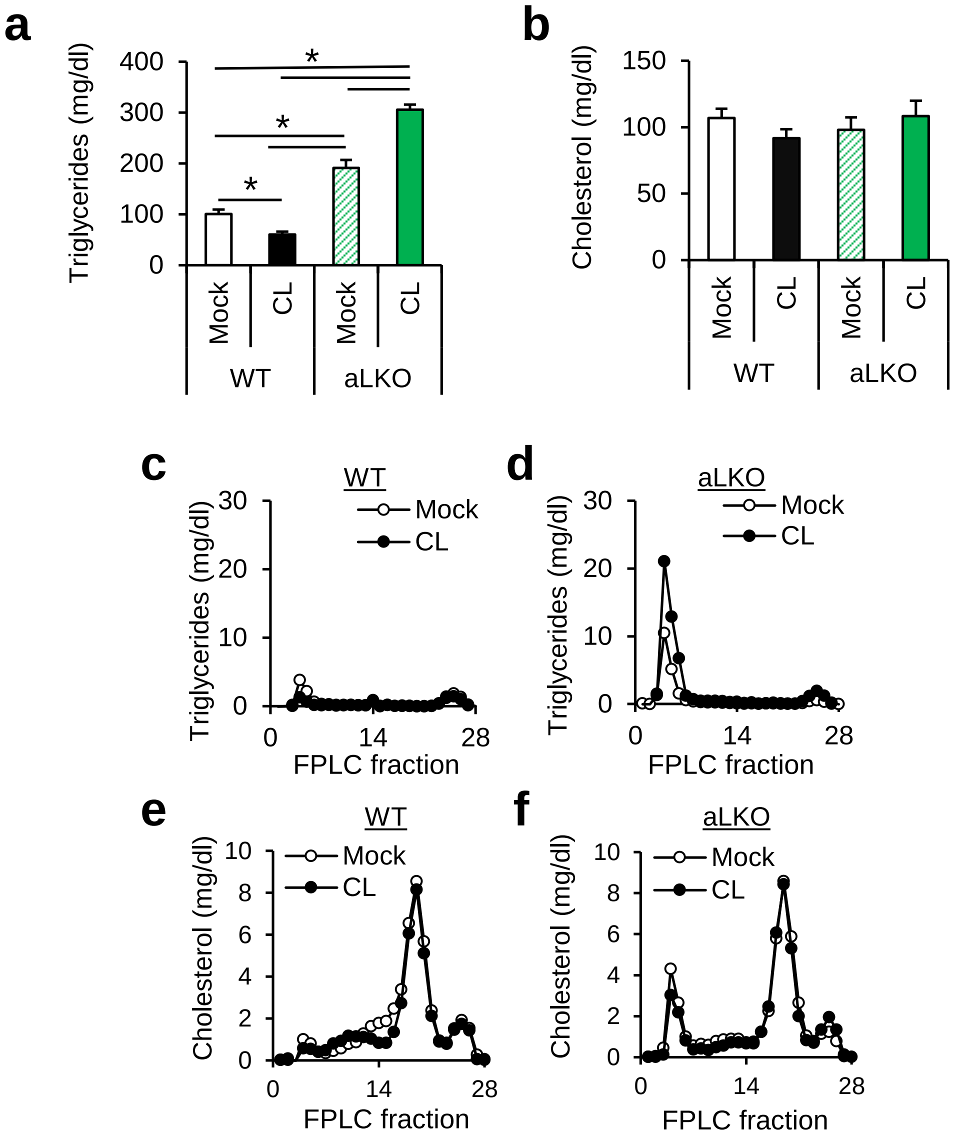

Supplementary Figure 9. LPL-dependent plasma lipid and lipoprotein profiles after thermogenic activation. Adipocyte-specific LPL knockout (aLKO) mice and wild-type littermate control (WT) mice were treated for 7 days with the β3-adrenergic agonist CL316,243 (CL) or saline (mock). Total plasma (**a**) triglycerides and (**b**) cholesterol levels. FPLC profiles of (**c, d**) triglycerides and (**e**, **f**) cholesterol of wild type (**c**, **e**) and aLKO (**d**, **f**) mice. Values are mean ± s.e.m. (n= 4 per group). *P<0.05 (2-way ANOVA). For FPLC analysis, plasma samples from each group were pooled.

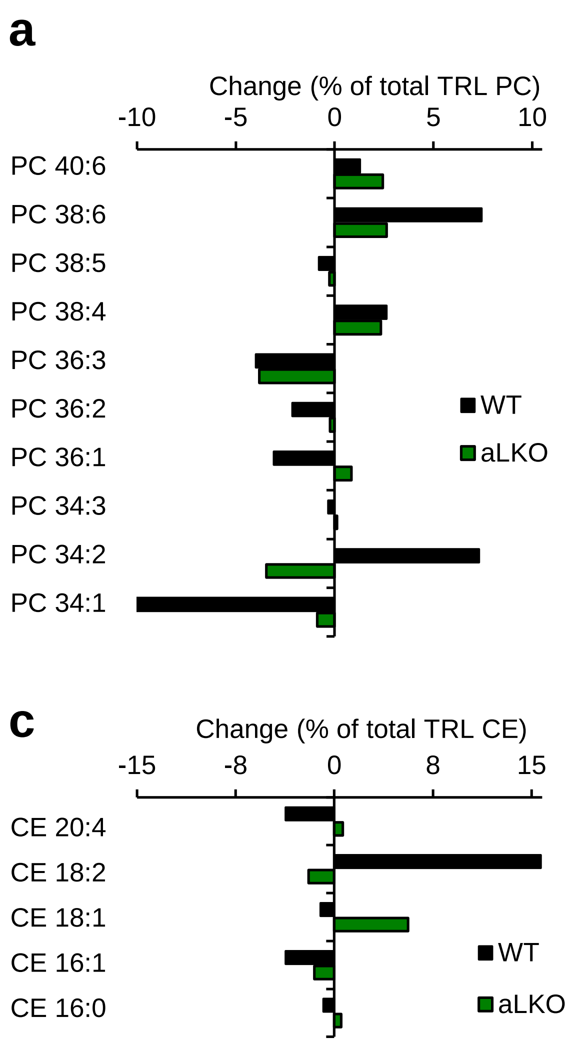

Supplementary Figure 10. Changes in TRL lipid composition induced by thermogenic activation are dependent on LPL. Adipocyte-specific LPL knockout (aLKO) mice and wild-type littermate control (WT) mice were treated for 7 days with the β3-adrenergic agonist CL316,243 (CL) or saline (mock). Lipids of TRL particles isolated by FPLC analysis (fractions 3-6; see supplementary figure 8) were determined MS-CID. Changes of (**a**) PC, (**b**) TG and (**c**) CE species are presented relative to mock-treated mice as change of % (weight) of total lipid class. For example, if a PC species has an abundance of 20% of total PC and increases to 25% of total PC after thermogenic stimulation, this is shown as +5%.

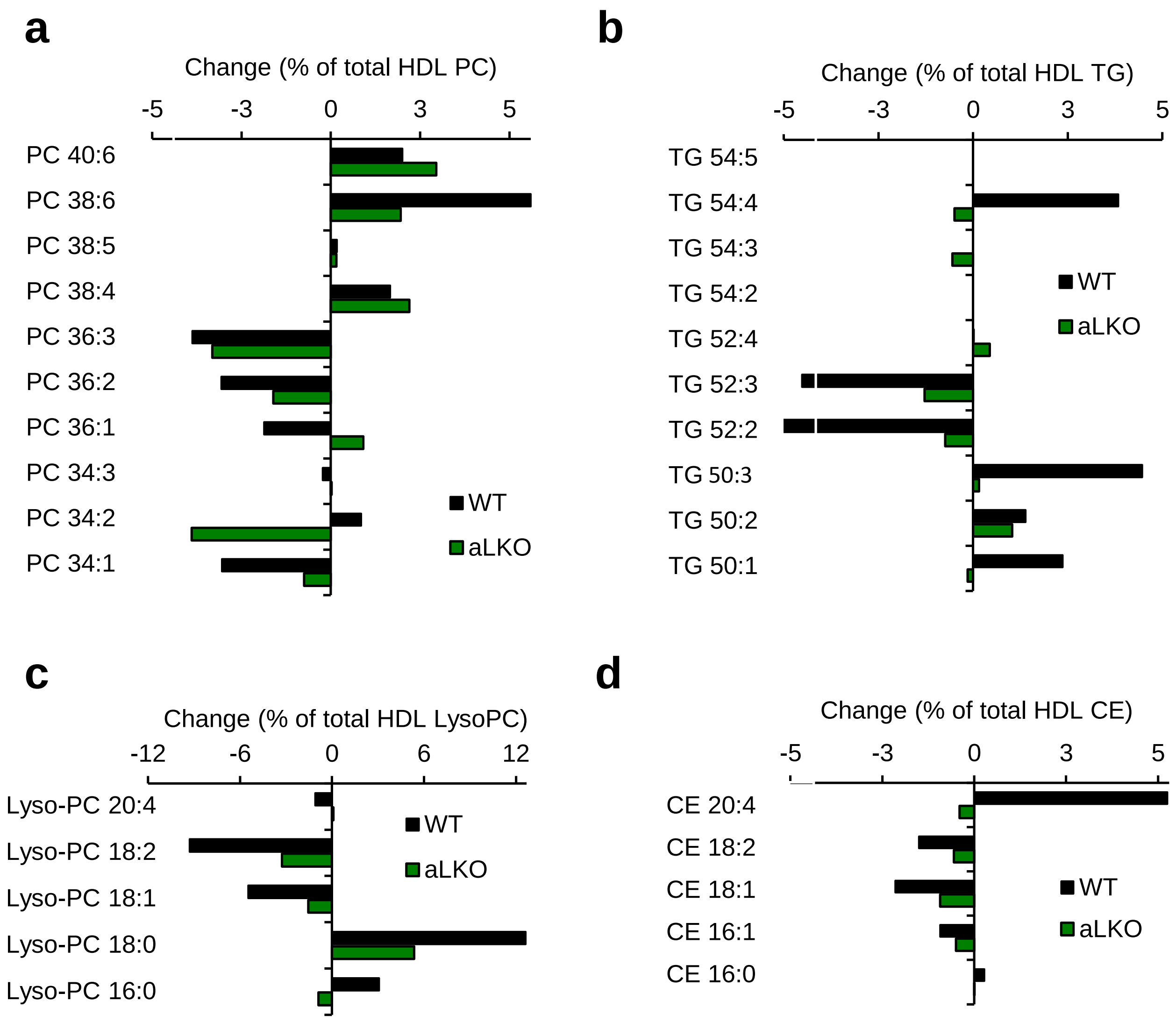

Supplementary Figure 11. Changes in HDL lipid composition induced by thermogenic activation are dependent on LPL. Adipocyte-specific LPL knockout (aLKO) mice and wild-type littermate control (WT) mice were treated for 7 days with the β3-adrenergic agonist CL316,243 (CL) or saline (mock). Lipids of HDL isolated by FPLC analysis (fractions 17-20; see supplementary figure 8) were determined MS-CID. Changes of (**a**) PC, (**b**) TG, (**c**) Lyso-PC and (**d**) CE species are presented relative to mock-treated mice as change of % (weight) of total lipid class. For example, if a PC species has an abundance of 20% of total PC and increases to 25% of total PC after thermogenic stimulation, this is shown as +5%.

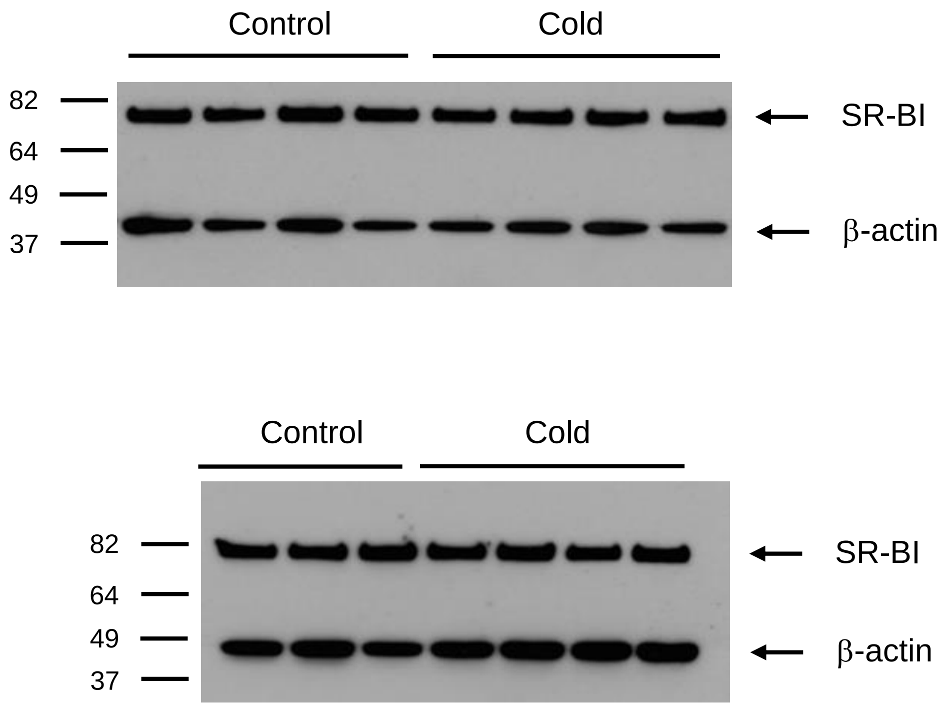

Supplementary Figure 12. Hepatic SR-BI expression is unaltered after cold exposure in wild type mice. C57BL/6 wild-type mice were housed at warm conditions (control) or exposed to cold for 7 days. Western blot of liver membrane fractions from two independent experiments are shown (n=3-4 per group). Molecular weights are given in kDa.

	Compound	Product Number	Supplier	Purity (%)	Concentration in extract (µg/ml)
1	1,2-Dipentadecanoyl-sn-glycero-3-phosphatidylcholine	P7285	Sigma aldrich	≥ 99	6.25
2	Cholest-5-en-3ß-yl heptadecanoate	110864	Avanti polar lipids	≥ 99	31.25
3	Glyceryltritridecanoate	T7517	Sigma aldrich	≥ 99	6.25
4	N-Heptadecanoyl-D-erythro-sphingosine	860517	Avanti polar lipids	≥ 99	6.25
5	1-Heptadecanoyl-2-hydroxy-sn-glycero-3- phosphocholine	110686	Avanti polar lipids	≥ 99	6.25
6	1,2-diheptadecanoyl-sn-glycero-3-phosphoethanolamine	110886	Avanti polar lipids	≥ 99	6.25
7	1-pentadecyl-3-(9Z-octadecenyl)-rac-glycerol	110593	Avanti polar lipids	≥ 99	6.25
8	1,3(d5)-Dipentadecanoyl-glycerol	110536	Avanti polar lipids	≥ 99	6.25
9	N-Heptadecanoyl-D-erythro-sphingosylphosphorylcholine	860585P	Avanti polar lipids	≥ 99	6.25
10	1,2-Di-O-tridecyl-sn-glycero-3-phosphocholine	999988P	Avanti polar lipids	≥ 99	6.25
11	1,2-Ditetradecanoyl-sn-glycero-3-phospho-L-serine	840033	Avanti polar lipids	≥ 99	6.25
12	1,2-Diheptadecanoyl-*sn*-glycero-3-phosphate	830856P	Avanti polar lipids	≥ 99	6.25
13	Heptadecanoic acid	H3500	Sigma aldrich	≥ 98	13.5

Suppl. Table 1: Constituent compounds serving as internal standards for high resolution lipidomic measurements

Lipid species	Fatty acid compostion *	Precursor ion (measured) *m/z*		Product ions (measured) *m/z*			
PC 40:6	18:0/22:6	$[M-H]^-$	832.5861	283.27	327.21		
PC 38:6	18:2/20:4	$[M-H]^-$	804.5548	303.22	279.22		
PC 38:5	20:4/18:1	$[M-H]^-$	806.5704	303.24	281.25		
PC 38:4	20:4/18:0	$[M-H]^-$	808.5861	303.24	283.24		
PC 36:3	18:1/18:2	$[M-H]^-$	782.5705	281.25	279.24		
PC 36:2	18:2/18:0, 18:1/18:1	$[M-H]^-$	784.5861	279.24	281.24	283.24	
PC 36:1	18:1/18:0	$[M-H]^-$	786.6018	281.25	283.27		
PC 34:3	no MS/MS	$[M-H]^-$	754.5392				
PC 34:2	18:2/16:0	$[M-H]^-$	756.5548	280.23	255.24		
PC 34:1	18:1/16:0	$[M-H]^-$	758.5705	281.25	255.24		
TG 50:1	16:0/16:0/18:1	$[M+NH4]^+$	850.7858	577.51	551.50		
TG 50:2	18:1/16:1/16:0, 18:1/18:1/14:0	$[M+NH4]^+$	848.7701	549.47	575.50	577.51	603.53
TG 50:3	18:1/16:1/16:1	$[M+NH4]^+$	846.7754	547.47	575.50		
TG 52:2	18:1/18:1/16:0	$[M+NH4]^+$	876.8014	577.51	603.53		
TG 52:3	18:1/18:1/16:1	$[M+NH4]^+$	874.7854	575.50	603.53		
TG 52:4	16:1/18:1/18:2	$[M+NH4]^+$	872.7701	601.51	573.48	575.50	
TG 54:3	18:1/18:1/18:1	$[M+NH4]^+$	902.8170	603.53			
TG 54:4	18:1/18:1/18:2	$[M+NH4]^+$	900.8014	601.51	603.53		
TG 54:5	18:1/8:2/18:2	$[M+NH4]^+$	898.7857	599.50	601.51		
TG 56:6	no MS/MS	$[M+NH4]^+$	924.8014				
TG 56:7	no MS/MS	$[M+NH4]^+$	922.7857				
TG 56:8	no MS/MS	$[M+NH4]^+$	920.7701				
TG 58:9	no MS/MS	$[M+NH4]^+$	946.7857				

* sn-position not determined

Suppl. Table 2: Fatty acid (FA) composition of lipid species esterified with more than 2 FAs
